# COVID-19 vaccination uptake and receptivity among veterans enrolled in homelessness-tailored primary health care clinics: provider trust vs. misinformation

**DOI:** 10.1186/s12875-023-02251-x

**Published:** 2024-01-12

**Authors:** June L. Gin, Michelle D. Balut, Aram Dobalian

**Affiliations:** 1 Veterans Emergency Management Evaluation Center, Department of Veterans Affairs, 16111 Plummer St. MS-152, North Hills, CA 91343 USA; 2 Division of Health Services Management and Policy, The Ohio State University College of Public Health, 202 Cunz Hall, 1841 Neil Ave, Columbus, OH 43210 USA

**Keywords:** COVID-19 vaccination, Vaccine hesitancy, Primary care, Homeless persons, Health behaviors, Health services research

## Abstract

**Background:**

Compared to the general population, individuals experiencing homelessness are at greater risk of excess morbidity and mortality from COVID-19 but have been vaccinated at lower rates. The U.S. Department of Veterans Affairs (VA)’s Homeless Patient Aligned Care Team (HPACT) program integrates health care and social services for Veterans experiencing homelessness to improve access to and utilization of care.

**Methods:**

This study explores the vaccination uptake behavior and attitudes through a qualitative comparative case study of two HPACT clinics, one in California (CA) and one in North Dakota (ND). Semi-structured telephone interviews were conducted with Veterans enrolled in the two VA HPACT clinics from August to December 2021 with 20 Veterans (10 at each clinic).

**Results:**

Four themes emerged from the interviews: (1) Vaccination uptake and timing— While half of the Veterans interviewed were vaccinated, ND Veterans were more likely to be vaccinated and got vaccinated earlier than CA Veterans; (2) Housing— Unsheltered or precariously housed Veterans were less likely to be vaccinated; (3) Health Care— Veterans reporting positive experiences with VA health care and those who trusted health providers were more likely to vaccinate than those with negative or nuanced satisfaction with health care; (4) Refusers’ Conspiracy Theories and Objectivity Claims— Veterans refusing the vaccine frequently mentioned belief in conspiracy theories while simultaneously asserting their search for objective information from unbiased sources.

**Conclusions:**

These findings amplify the importance of improving access to population-tailored care for individuals experiencing homelessness by reducing patient loads, expanding housing program enrollment, and increasing the provider workforce to ensure personalized care. Health care providers, and housing providers, social workers, and peers, who offer information without discrediting or criticizing Veterans’ beliefs, are also key to effectively delivering vaccine messaging to this population.

**Supplementary Information:**

The online version contains supplementary material available at 10.1186/s12875-023-02251-x.

## Background

Individuals experiencing homelessness face elevated risks of mortality, infection, and adverse health impacts from SARS-CoV2, the virus that causes COVID-19, due to their reliance on crowded congregate settings for shelter and other services [1, 2], elevated rate of health conditions [3–6], and barriers to health care [4, 6]. However, individuals experiencing homelessness in the US have received the recommended vaccine for COVID-19 at lower rates than the general population [7–12], further amplifying their risk. The factors depressing vaccine uptake among unhoused individuals remain poorly understood; prior studies have alluded to vaccine hesitancy, mistrust of authorities, lack of access to health care, and competing priorities as potential factors hindering vaccine uptake within this population [5, 13–19]. Traumatic experiences related to homelessness, such as stigma and racism in health care settings, may make these individuals especially susceptible to misinformation around COVID-19 vaccines, further discouraging vaccine acceptance [16, 20].

Health care access and utilization have been shown to improve health outcomes and adherence to disease management among individuals experiencing homelessness [3, 21–23]. Specifically, Veterans experiencing homelessness have benefitted from health care models tailored to them, which can help reduce feelings of stigma and enhance trust in care providers [24–27]. Reducing stigma and increasing primary care visits with trusted health care providers have been linked to COVID-19 vaccine acceptance among Veterans enrolled in U.S. Department of Veterans Affairs’ (VA) Homeless Programs [13, 28]. Understanding the vaccination behavior and attitudes of Veterans enrolled in homeless-specific health care is key to vaccine adoption.

### VA Homeless Patient Aligned Care Team (HPACT) programs

Population-tailored health care models have been shown to increase primary care visits [29], reduce acute services use [30, 31], and provide better care experiences among homeless patients [29, 32–34]. The VA has established a multitude of programs to address the unique and complex needs of Veterans experiencing homelessness. Since its launch in 2012, 54 sites have implemented Homeless Patient Aligned Care Teams (HPACTs) in VA Medical Centers (VAMC), Community-Based Outpatient Clinics (CBOCs), and Community Resource and Referral Centers (CRRCs). HPACTs are multidisciplinary primary care teams consisting of physicians, nurses, social workers, and mental health counselors who coordinate medical care with mental health and substance abuse treatment and housing placement [24]. The VA provides housing to Veterans through VA and multiagency homeless programs, including in conjunction with the U.S. Department of Housing and Urban Development (HUD) through VA Supportive Housing (HUD-VASH), which provides subsidized vouchers for rental assistance for eligible Veterans to maintain permanent housing [35], and through the VA Grant and Per Diem (GPD), which issues grants to nonprofit organizations providing transitional housing and social services to Veterans [35]. GPD programs have been found to help reduce barriers and facilitate COVID-19 vaccination access and uptake for enrolled Veterans [7, 14].

VA HPACT programs have provided a much-needed bridge connecting Veteran participants to needed care, including chronic disease management and mental health [26], social support to help manage the daily challenges of homelessness (e.g., housing, food, transportation), and a stigma-free, easy-to navigate care setting structured to accommodate their needs [24, 25]. Staffed with providers specializing in care for Veterans experiencing homelessness, HPACTs are uniquely positioned to engage in COVID-19 vaccination efforts for this population [13]. This study explores HPACT Veterans’ behavior and attitudes toward COVID-19 vaccination.

## Methods

### HPACT sites

The study population consisted of Veterans receiving care from one of two VA HPACT programs. One HPACT clinic was in California (CA) and consisted of 13 clinical teams serving approximately 2,600 Veterans. HPACT Veterans were recruited from three clinical teams from two VAMC care centers and one CBOC, serving a combined population of 1,052 Veterans. The other HPACT clinic, in North Dakota (ND), was a small, relatively new program housed in a CRRC, and serves 110 Veterans.

### Recruitment & data collection

Veterans were invited to participate by their HPACT health care providers who collected Veterans’ names and phone numbers and, with their consent, shared that information with the researchers, who then contacted the Veterans directly. HPACT health care providers at the two sites were also interviewed to learn about their experiences during COVID-19 and their efforts to facilitate Veterans’ vaccination; these perspectives are reported elsewhere [13, 36].

From August to December 2021, the first and second authors conducted semi-structured telephone interviews lasting 30–60 min with Veterans enrolled in the two HPACT clinics in California (n = 10) and North Dakota (n = 10). Interviews were conducted until data saturation was reached [37], resulting in 20 Veteran interviews out of 36 Veterans who were contacted. Researchers obtained verbal consent from participants before study inclusion. The only inclusion criterion was that all participants were Veterans enrolled in one of the two HPACT clinics. Each Veteran was given a $10 incentive as compensation for their study participation.

The interview guide was developed for this study by the researchers and consisted of 20 open-ended questions (see supplementary file 1). Participants were asked their opinions about the COVID-19 vaccine, their reasons for their decisions regarding whether to get vaccinated, whether they hesitated or delayed vaccination, and the factors behind those delays. They were also asked whether they were worried about COVID-19, their sources of information about the pandemic and the vaccines, experiences with health care and trust in health care providers, general vaccine views, and sociodemographic characteristics.

### Data analysis

All interviews were audio recorded, transcribed, and analyzed using the rapid analysis approach [38, 39]. Findings were analyzed thematically, both through inductive grounded theory [40] focusing on themes that emerged during the interviews and through a priori deductive themes organized in a summary table document by key domains based on the interview guide. Using the summary table template, the transcripts were divided and summarized by two team members, grouping information by shared content. The summary document was modified to reflect new inductive themes that emerged during analysis. Summaries were then unified into a single, high-level document to identify commonly occurring themes across interviews. The substantive significance of themes, the extent and context in which these themes were present in the data [41], was used to identify significant findings.

This article utilized a comparative case study approach to examine vaccination attitudes and behavior of the two groups of HPACT Veterans, which enabled the exploration of similarities and differences to identify factors that may shape different outcomes, as well as aspects common to both groups. Such comparisons between groups can facilitate “both discovery and theory development” [42], through the “completeness and consideration of alternate perspectives” [42]. Incorporating grounded theory approaches into comparative case studies facilitated the identification of common themes that ran across the cases, strengthening the evidence [43].

## Results

Four themes describing differences between ND and CA Veterans’ COVID-19 vaccination emerged from the interviews: (1) COVID-19 vaccination outcomes, timing, and attitudes; (2) housing status; (3) health care experiences; and (4) vaccine refusers’ beliefs in conspiracy theories and their claims of informational objectivity. Table 1 illustrates characteristics of the 20 Veterans who participated in this study, organized by site, including COVID-19 vaccination status, self-identified race/ethnicity, age, worry about COVID-19, self-identified medical risk, housing status, satisfaction with HPACT health care, receipt of COVID-19 vaccine information from HPACT, influenza vaccination uptake, and belief in conspiracy theories. All Veteran respondents were male, ranging in age from 22 to 75 years old, with most in their 50 and 60 s. CA Veterans tended to be older than ND Veterans, and more likely to be people of color—five identified as African American and two as Hispanic, while one declined to state his race. Among ND Veterans, seven identified as White and three as African American.

**Table 1:** Vaccination Outcomes and Characteristics of HPACT Veterans

Outcomes and Characteristics		Overall N = 20 No. (%)	CA N = 10 No. (%)	ND N = 10 No. (%)
COVID-19 Vaccination Status	Vaccinated	10 (50)	3 (30)	7 (70)
	Not vaccinated and hesitant	8 (40)	5 (50)	3 (30)
	Delay- unvaccinated but willing	2 (10)	2 (20)	0 (0)
Race/Ethnicity	White	9 (45)	2 (20)	7 (70)
	African American	8 (40)	5 (50)	3 (30)
	Hispanic	2 (20)	2 (20)	0 (0)
	Declined to state	1 (10)	1 (10)	0 (0)
Age (years)	< 29	2 (10)	1 (10)	1 (10)
	30–49	4 (20)	1 (10)	3 (30)
	50–69	13 (65)	7 (70)	6 (60)
	70+	1 (5)	1 (10)	0 (0)
Worried about COVID-19	Yes	6 (30)	2 (20)	4 (40)
	No	14 (70)	8 (80)	6 (60)
Medically At-Risk	Yes	7 (35)	4 (40)	3 (30)
	No	12 (60)	5 (50)	7 (70)
	Unsure	1 (5)	1 (10)	0 (0)
Housing Status	HUD-VASH or own apartment	10 (50)	3 (30)	7 (70)
	Transitional housing (incl GPD)	4 (20)	1 (10)	3 (30)
	“Tiny Shelters” at CA VA	2 (10)	2 (20)	0 (0)
	Unsheltered	4 (20)	4 (40)	0 (0)
Satisfied with HPACT Health Care	Yes	16 (80)	7 (70)	9 (90)
	No	4 (20)	3 (30)	1 (10)
Received Information about Vaccines from HPACT	Yes	12 (60)	4 (40)	8 (80)
	No	4 (20)	2 (20)	2 (20)
	There were gaps	4 (20)	4 (40)	0 (0)
Influenza Vaccination Status	Vaccinated	9 (45)	4 (40)	5 (50)
	Not vaccinated	11 (55)	6 (60)	5 (50)
Expressed Belief in or Mentioned Conspiracy Theories	Yes- mentioned No- but mentioned No- didn’t mention	10 (50) 3 (15) 7 (35)	5 (50) 1 (10) 4 (40)	5 (50) 2 (20) 3 (30)

### COVID-19 vaccination outcomes, timing, and attitudes

Half of all HPACT Veterans who participated in this study were vaccinated for COVID-19 by December 2021. Vaccinated Veterans spanned multiple race/ethnicity and age categories, however more of the ND Veterans were vaccinated than their CA counterparts (seven vs. three). An additional two Veterans in CA expressed a willingness to get vaccinated but had not done so by the time of their interview- one was waiting to get vaccinated until after a scheduled surgery while the other was initially hesitant but was planning to get vaccinated the day of the interview due to concerns over the Omicron variant:

*“I’ve been hearing about the new variant that’s coming out, and they said that it’s more—you can catch it quicker or easier with the new variant. So I just decided to just go ahead and get the vaccine…” (CA19)*.

However, Veterans in both sites expressed opposition to and distrust of the vaccine, including those who ultimately received the vaccine. Most refusers stated they did not trust the vaccine’s safety and effectiveness. A few said they did not believe they needed it, because they took precautions to avoid infection, because they did not view themselves as being at risk despite, in some cases, having medical comorbidities such as high blood pressure or diabetes, or because they believed that COVID-19 had been “overhyped” by the media and public officials. One Veteran explained that getting the vaccine was not a priority given the competing demands of daily life under conditions of homelessness. Veterans who received the vaccine despite their distrust said they either “wanted to get it over with” because they believed that access to jobs, housing, or entry to venues would be contingent on vaccination, or were successfully persuaded by health care providers or peers to overcome their distrust and take action to protect their health. Three CA Veterans were vaccinated, while two were willing to vaccinate but had not done so by the time of the interview, suggesting that there were still receptive Veterans who were still unvaccinated 8–10 months after the vaccine became available at the VA.

There were also significant differences in Veterans’ reported vaccination timing and vaccine hesitancy attitudes between the CA and ND Veterans. Four of the seven vaccinated ND Veterans reported wanting the COVID-19 vaccine as soon as it was available, and by May 2021, five of the seven had already received it, with the remaining two delaying until that summer due to feelings of mistrust and hesitancy. Three ND Veterans received their vaccines at two “vaccination blitzes” HPACT providers hosted in their clinic in February 2021, facilitating easy and early COVID-19 vaccine access. Some ND Veterans had their VA HUD-VASH caseworker drive them to their vaccination appointment. In contrast, even CA Veterans who did get vaccinated described a lengthy process of deciding to get the vaccine and then trying to figure out how to achieve that goal. Only one of the three vaccinated CA Veterans received the vaccine in March 2021 shortly after it was available. The other two vaccinated Veterans described feelings of hesitancy, including worries about the newness of the vaccine and potential side-effects, as reasons for delaying and only accepted the vaccine in August and October 2021, respectively, after months of verbal persuasion efforts from VA physicians and friends (see Health Care Experiences theme below). Two additional CA Veterans remained unvaccinated in September and December 2021, respectively, despite expressing vaccine willingness. CA Veterans typically relied on transit or private vehicles to transport them to vaccine appointments and faced logistical and motivation barriers. Overall, less vaccine hesitancy than CA Veterans, which, along with convenient access at the highly publicized vaccination blitzes, likely facilitated their earlier receipt of the vaccines, and possibly higher vaccination rates among their cohort overall.

Veterans also expressed varying levels of worry about contracting and becoming ill from COVID-19, with those reporting being very worried often taking action by getting vaccinated. Vaccinated Veterans, particularly in the ND HPACT, were less worried after vaccination, and even those who were not worried described taking precautions to avoid infection. In contrast, vaccine refusers were unanimous in reporting not being worried at all. Reasons for not being worried included believing that precautions like social distancing would naturally offer safety, stating they had immunity from prior COVID-19 infections or believing COVID-19 does not exist. Additionally, Veterans in CA who self-identified as medically at-risk for COVID-19 reported being worried about COVID-19 and seemed receptive to vaccination. In contrast, ND Veterans seemed open to vaccinating in higher numbers regardless of medical risk. However, ND6 decided to get vaccinated, and was willing to get a booster, because of his multiple medical conditions, likely a result of experiencing chronic homelessness:

*“Well, they told me I’m at high risk for drug addiction, homelessness, I have severe depression, anxiety. I got blockage in both of my legs just below my knees… and I’m a throat cancer survivor… I’ve also had tuberculosis… and then I had hepatitis C. So, I’ve been through the wringer the last few years… so I said, yeah, I better go ahead and take those shots just in case… I’d be willing to take that booster because [of] that new variant. I just don’t wanna get this. I’ve been through enough. I don’t need to add to my growing list of things I’ve been through.”* (ND6).

These differences in vaccine attitudes and acceptance rates between the ND and CA Veterans raised questions about other similarities and differences that could potentially impact vaccine uptake.

### Housing status

ND Veterans were much more likely to be stably housed than CA Veterans, who were more likely to be unsheltered homeless. At the CA site, four Veterans were unsheltered—living in cars or outdoors—and two others were residing in a “tiny shelter village” through the CA VAMC’s Care, Treatment, and Rehabilitative Service (CTRS) located outdoors on the VA campus, a low-barrier program that allows unhoused Veterans to enroll without mandated sobriety or other qualifications. Additionally, one lived in transitional housing through the VA GPD program and 3 had their own permanent housing through HUD-VASH. In contrast, seven ND Veterans had their own housing through HUD-VASH, and three lived in transitional housing. All but one ND Veteran in the HUD-VASH program had lived in their own housing with vouchers for years. These patterns mirror overall Veteran homelessness statistics for their respective regions: where the CA site is located, 78.7% of homeless Veterans are unsheltered, as compared to only 2.7% of homeless Veterans unsheltered in ND [44].

CA Veterans who were unsheltered or living in tiny shelters were more likely to be unvaccinated, representing four of the five vaccine refusers. A few of these Veterans noted that living in unsheltered conditions made vaccination seem less urgent given competing priorities of survival. CA17, who was living in the CA VAMC’s outdoor “tiny shelter village”, noted that despite living in a communal environment, he was seeing very little evidence of the pandemic:

*“I’m in an area where COVID-19 should exist. …using porta johns, using showers after people…. It’s cold here, it’s wet, it’s damp, there are rats, there’s fungus, unwashed people. I’m in a high-risk area…we don’t eat properly. The food here is non-nutritional, so I know my body is not well-defended against sickness…and that’s why I feel like COVID should be rampant through here, and we get tested every two weeks, and I haven’t seen anyone get taken away by ambulance or taken out for COVID.” (CA17)*.

This narrative illustrates how, for people experiencing unsheltered homelessness, COVID-19 may not have “felt real” because it was not confirmed by the reality of their everyday lives. CA Veterans who delayed vaccination but were willing were also currently or recently unsheltered. Of the three CA vaccinated Veterans, only one was unsheltered while two had been vaccinated while living in GPD housing, pointing to such programs’ roles in stabilizing Veterans’ everyday lives, meeting their urgent competing needs such as food and shelter, and facilitating both education about the vaccine and logistical aspects of their vaccination. CA Veterans who were unvaccinated had more precarious housing conditions in 2021, the first year of vaccine availability, which may have hindered their access to vaccination opportunities and information.

In ND, four of the seven vaccinated Veterans had been living in HUD-VASH funded housing for several years, and the remaining three lived in transitional housing. Housing status did not differ substantially for the three unvaccinated ND Veterans, although ND1, a vaccine refuser, had only moved into a HUD-VASH apartment in the prior week after living unsheltered for several months since his military discharge. He stated that he was not worried about COVID-19 and not interested in getting vaccinated because he has other pressing concerns:

*“I was homeless. I wasn’t worried about [COVID-19] at all. I have bigger problems… If you don’t know where you’re gonna sleep, nothing else is a priority besides knowing where you’re gonna sleep or where you’re gonna get your food at… Vaccines, medications, none of that is a priority. If you don’t know where you’re gonna sleep or eat, nothing else matters.” (ND1)*.

Overall, living unsheltered seemed to exacerbate vaccine hesitancy and other vaccination barriers, among Veterans experiencing homelessness, pointing to the role of housing and stable shelter in facilitating vaccination by reducing their burden of competing survival needs.

### Health care experiences

The HPACT clinics were especially important to providing convenient access to care among unhoused Veterans, and by extension, facilitating their vaccination access. Both HPACT clinics had downtown locations situated near housing programs and other services for people experiencing homelessness. For example, the ND HPACT’s vaccination blitz in February 2021 was intended to reduce perceived vaccination barriers. Overall, Veterans’ VA health care experiences, particularly with HPACT providers, seemed to shape their vaccination decisions and behavior. Not only did ND Veterans praise the quality of the health care they received at the HPACT clinic, but they also cited the deep trust they had for the HPACT staff as key factors in their decision. Half of the ND Veterans volunteered that the ND VA overall provided higher quality care and service than other health care systems—including two who mentioned worse experiences at VAs in other states, and three who had previously received substandard health care in prison, the military, and a non-VA health care site, respectively. In ND, nine out of 10 Veterans described positive experiences at the HPACT, and several complimented the clinic’s primary care provider, whom they described as empathetic and caring:

*“I’ve gone through eight doctors in the years that I’ve been at the VA and [HPACT provider] has… has been one of the better ones I’ve ever had… she’s on the top 10 list with me. I trust her. I feel that she’s very educational. She’s very caring. She’s got a lot of empathy… And that makes me feel comfortable and it makes other people feel comfortable. When you have somebody that is kind, honest, you know, treats you with respect, you can’t beat it.” (ND5)*.

Four ND Veterans, all of whom were vaccinated, said they had also received excellent care from the main VA before switching to the newly opened HPACT clinic. However, two out of the three ND Veterans who refused the vaccine reported dissatisfaction and bad experiences at the main VA prior to enrolling in HPACT, which may have contributed to their stated distrust of health care overall.

The majority of CA Veterans also reported being satisfied with HPACT health care, including two of the five vaccine refusers. However, their VA experiences were more nuanced. CA13, a longtime VA user who eagerly received the vaccine immediately upon availability, noted that while he is pleased with his VA health care, it was only in the past decade that the quality of care began to improve. CA5, who was still unvaccinated but otherwise willing, enumerated a lengthy list of miscommunications, inefficiencies, and other barriers he encountered while seeking VA care despite concluding that he found VA health care to be a positive experience overall. Finally, three of the five vaccine refusers reported dissatisfaction and bad experiences at the VA, however, two were relatively new to HPACT.

Differences between these two groups’ health care quality perceptions carried over into their narratives about the VA health care providers’ role in recommending the COVID-19 vaccine. In ND, eight out of 10 Veterans said that their HPACT health care provider directly spoke with them about the vaccine’s benefits and recommended that they get vaccinated. Four ND Veterans were eager to get the vaccine and said they did not need any persuading, but five of the seven vaccinated Veterans and all three vaccine refusers nonetheless reported such provider discussions, suggesting extraordinary thoroughness in health care messaging. The two ND Veterans who reported receiving no verbal VA messaging both eagerly received their vaccines at the “blitz”. It is possible that given their high vaccine enthusiasm, providers may not have felt it necessary to provide information or the Veterans did not perceive their dialogue as a form of messaging. ND1, a vaccine refuser, noted that provider messaging about the vaccine would not make a difference because he distrusted doctors and health care in general.

The CA HPACT had a more challenging experience. Only four of 10 CA Veterans reported VA health care providers speaking with them about the vaccine and recommending it. However, three Veterans who were vaccinated mentioned VA health care providers’ repeated efforts over the course of several months to persuade them to accept the vaccine. In the absence of these conversations, they would still be unvaccinated, they said:

*“It took the longest for me to make a decision to take the vaccine, I was very apprehensive about it, and I heard so many stories and stuff, you know, because I really didn’t know, my doctor told me that once I [was vaccinated] that there was a slim possibility that I would get [COVID-19] again. So I listened to my doctors… he took time out to, really out of concern… he really appealed to me, so I got the vaccine… he sounded very concerned. And I thank him for that… I’ve been knowing him for years and I’ve been on that unit before, he’s always treated me with respect, and he’s been very candid, and I appreciated that, and I got good treatment on that unit. And I have a lot of respect for him.” (CA8)*.

CA8 mentioned that it was not the VA HPACT, but rather a VA physician who had previously treated him in a VA-inpatient clinic, who successfully persuaded him to get vaccinated. CA19, who was unvaccinated but willing, also mentioned a VA physician convincing him to schedule his vaccine in December 2021. These three Veterans’ lengthy delays in vaccinating illustrate the extraordinary patience and perseverance required from VA providers in persuading their patients to accept the vaccine. Given the vastly larger size of the CA VAMC, such individualized time and attention to patients may have been difficult for clinicians to achieve.

Four CA Veterans experienced gaps when receiving information about the vaccines from the VA and two did not receive any information at all. CA5, the unvaccinated but willing Veteran, claimed that he was not contacted when the vaccine was initially available, and recently, VA providers had given him printed vaccine information, but he was unable to read it due to vision problems. CA5 claimed a lack of coordination of care when transferring from a non-VA clinic into the VA HPACT, as well as transportation and other logistical challenges he had to navigate. However, he noted that these issues are likely due to the VAMC’s large size and patient caseload:

“*Nobody mails you anything or calls you from the VA… and that’s nothing bad…the VA’s so big… they probably have a million other more problems than that… maybe… because it’s a big city, and VA’s a big place here… and they got a lot of other problems they got to deal with too*.*” (CA5)*.

VA providers had begun offering him the vaccine in the Summer/Fall of 2021, but he noted that they were not aware of his medical history, including surgeries, that caused him to be fearful of vaccination. Three CA Veterans who received VA information with less than a full endorsement were all vaccine refusers. They mentioned that their health care providers had offered the vaccine but stopped short of recommending it. “*He said ‘it’s up to you’”* (CA16 and CA17) was their shared refrain. Of the two who did not receive vaccine information, one was vaccinated, and one refused. CA13, the vaccinated Veteran, noted that HPACT providers likely did not talk to him about the vaccine because he regularly requested it prior to its availability. The other, CA18, a vaccine refuser, expressed distrustful views toward the VA, saying that he refuses government information.

Veterans were asked which sources of information about the vaccine they trusted the most. Trust in the VA was high- over half of the Veterans at both locations mentioned trusting information from the VA or VA health care providers about the vaccine and COVID-19, consistent with Veterans’ high level of satisfaction with their respective HPACT clinics. Notably, even vaccine refusers from both sites—one from ND and three from CA—said they relied on and trusted the VA and health care providers for vaccine information. The health care system, whether it was their own provider, the VA, or another health care professional (e.g., prison hospital, pharmacy), was mentioned as sources of information by over half of the Veterans at both sites (six ND Veterans and six CA Veterans).

Veterans were also asked about their uptake of the influenza (flu) vaccine, and their attitudes toward non-COVID-19 vaccines in general. In both ND and CA sites, all vaccine refusers reported not receiving a flu shot in the past two years, some reporting an opposition to all vaccines while others provided more nuanced perspectives, saying that they were not against all vaccines, but believed that vaccines for the flu and COVID-19 were unnecessary or ineffective. Two of the seven vaccinated ND Veterans did not receive flu shots, but they both noted that they had received other vaccines, for Hepatitis A and shingles, respectively, while the remaining five vaccinated Veterans also received the flu shot. All three vaccinated CA Veterans and CA5, the unvaccinated but willing Veteran, received the flu shot and were strongly pro-vaccine, while the remaining unvaccinated but willing Veteran did not receive the flu shot.

### Vaccine refusers’ beliefs: conspiracy theories and claims of informational objectivity

Among Veterans who refused the vaccine, two themes were mentioned frequently as they described their decision-making around vaccination: conspiracy theories and claims of independence and objectivity in information-seeking. “Conspiracy theories”, commonly defined as explanations for important events that involve secret plots by powerful and malevolent groups [45], have been pervasive throughout the COVID-19 pandemic, and have been found to suppress vaccine uptake by fueling skepticism and distrust of entities promoting vaccination [16, 20, 46–48]. While conspiracy theories are related to distrust surrounding the vaccine, these narratives often contained allegations of deliberate and malevolent deception or effort by government, media, or pharmaceutical industry to conceal dangers from the public. For the purposes of this analysis, “conspiracy theories” are distinct from expressed distrust only due to uncertainty or the newness of the vaccine. According to these Veterans’ narratives, they could not trust government nor the media, forcing them to “do their own research” to find “unbiased” information. These were emergent themes—neither solicited nor prompted as part of the interview, wherein Veterans were asked to describe how they made their vaccination decisions and their information sources.

Conspiracy theories strongly emerged in vaccine narratives, mentioned by 13 of the 20 Veterans. However, only 10 of the Veterans expressed their own beliefs in such ideologies, while 3 did not hold these beliefs but cited their perception of the pervasiveness of conspiracy theory beliefs in their peer group. “Conspiracy theories”— ranging from COVID-19 and vaccine dangers to global collusion to consolidate power and engage in population control—emerged both in interviews with vaccine refusers and vaccinated Veterans. In both CA and ND, all vaccine refusers expressed beliefs in perspectives that we identified as “conspiracy theories”. These conspiracy theories were not expressed by any the vaccinated nor the willing CA Veterans, other than complaining about others being influenced by conspiracy theory beliefs, suggesting that their delays in vaccinating were not fueled by mistrust. However, three vaccinated Veterans in ND also expressed their belief in COVID-19 conspiracy theories, while a fourth vaccinated Veteran noted that many of his peers refuse the vaccine because of misinformation, suggesting that such views were pervasively circulating within the ND environment and among peers. In addition to common anti-vaccine narratives (e.g., “*it does something to your DNA*” [CA17]), Veterans described personal experiences of feeling deceived and mistreated by government entities. One vaccine refuser who suffered from PTSD shared his belief that VA physicians had medicated him and framed him for killing fellow Marines in combat, causing him to be stripped of his combat Veteran designation:

*“People sending me letters telling me I’m not a combat Veteran….head of the [VA] PTSD program, he believed the people who were lying. …he did something that he was not supposed to do, he talked about me to my probation officer and said I was the Antichrist.” (CA15)*.

Vaccine refusers expressed their belief in conspiracy theories while simultaneously claiming to be independent and objective thinkers in their search for information. They described their claims of informational objectivity as “critical thinking”, often criticizing others for spreading fear-based conspiracy theories or biased information. Additionally, two vaccine refusers (one at each site) mentioned conservative “experts” on YouTube, and two CA vaccine refusers (CA16 and CA18) were skeptical of all sources, instead trusting their “own intuition”. For example, one vaccine refuser blamed media outlets for spreading fear-based conspiracy theories:

*“There’s a lot of tin foil hats that have folks terrified to do anything. One of them is [a political commentator] on Fox News. He has a lot of folks terrified to take anything. So, when you listen to the news, they tell you, you should be afraid of this because this and that. So, if there were more information about it that was unbiased…they’re trying to just tell me where and when and what I should do and why, I wouldn’t have an issue.” (ND1)*.

Despite his claim of objectivity, he was affected by these conspiracy theories, citing his own fears leading him to refuse the vaccine:

*“I don’t wanna be the guy calling some lawyer at 69 because they have fertility problems because of the COVID shot or something or anything like that” (ND1)*.

This defensiveness was common among vaccine refusers who believed in conspiracy theories. Refusers were more assertive than vaccinated Veterans about citing official government sources, such as the U.S. Centers for Disease Control and Prevention (CDC), the Vaccine Adverse Event Reporting System (VAERS) database, and the VA, when asked about their sources of information about the vaccine. Vaccinated Veterans, in contrast, were likelier to cite the news, their own health care providers, and word-of-mouth. CA16, a vaccine refuser, described critically evaluating information sources:

*“I don’t like to just go with one source. Ever since vaccines have been available, I’ve been reading about what they are…. leading chemists, what the company says about them, I try to get as much information as I possibly can….Doing research is something that—it’s a double-edged sword. It can confuse you and give you the wrong opinion of everything. So until I really kind of find a unanimous opinion about…the vaccine, I try to keep my mind open…”* (CA16).

Vaccine refusers at both sites had similar narratives of “*doing one’s own research*” or “*critically evaluating multiple information sources*”, asserting that they did not passively accept information. However, rather than receiving objective, accurate information from these sources, they interpreted government information in ways that supported their own suspicions of harm attributed to the vaccine:

“*The VAERS thing, the vaccine reporting thing, this vaccine has killed more people in just the last year than since the reporting system has been valid. And it’s harming people right and left. People are dying a day or two after they get the shot*.” (ND13).

They were highly aware of the stigma around conspiracy theories. Several refusers who believed these theories addressed the label directly, asserting that they were not conspiracy theorists:

*“My perception of everything that’s gone on in the last… 50 years… I’m seeing the monetary system taken over, I’m seeing a lot of control being brought about. That seems a conspiracy theory type of viewpoint, and I very much try not to lean on that train of thought, of conspiracy theory type of thing…”* (CA16).

*“And in the trials when they did [testing], they killed every single animal that it was tested on…and I’m not one of these conspiracy theorist guys, but, when it’s a proven fact it’s not very much a theory anymore. It’s a conspiracy fact*…” (ND13).

The ubiquity of conspiracy theories in Veterans’ perspectives offers insight into factors driving vaccine refusal. Along with other themes such as health care experiences, pandemic attitudes, and housing status, these data paint a portrait of the typology of Veterans who may resist vaccines. Notably, conspiracy theory beliefs were found in both vaccinated and refusing Veterans in ND, whereas only CA vaccine refusers expressed beliefs in conspiracy theories.

## Discussion

People experiencing homelessness [9, 11, 12] and Veterans experiencing homelessness specifically [7] have lower COVID-19 vaccination uptake rates than the general U.S. population. However, existing research [13, 14, 49] has shed limited light into the myriad factors underlying these depressed vaccine uptake rates, as quantitative research may not fully reveal complex patterns in Veterans’ behavior and attitudes. Half of the HPACT Veterans in this study were vaccinated, consistent with findings from similar studies [7, 9, 11]. In addition, Veterans at the CA HPACT site had a lower vaccination rate than those at the ND HPACT (30% vs. 70%, respectively). In analyzing vaccination behaviors, attitudes, and uptake among Veterans at two HPACT sites with very different characteristics, this study found noteworthy variation between the two sites, providing insight into factors driving vaccine behavior.

The ND HPACT achieved significantly higher vaccine uptake than the CA site despite the disadvantage of a seemingly less favorable political environment where conspiracy theories and misinformation were prevalent. As a state, ND is widely viewed as being more politically conservative than CA, which has been associated with greater COVID-19 vaccine resistance [50]. Nearly equal numbers of Veterans in ND and CA believed conspiracy theories; however, three ND Veterans who believed in conspiracy theories got vaccinated, whereas the CA HPACT were unable to persuade hard-core refusers to get vaccinated. There are a few possible explanations for ND Veterans accepting the vaccine in higher numbers despite the widespread belief in conspiracy theories. Early and convenient access to the COVID-19 vaccine, which has demonstrable benefits for improving uptake [10, 13, 51, 52], likely facilitated ND Veterans’ higher vaccination rates. Two ND Veterans who believed in conspiracy theories received their vaccines at the January 2021 blitz. The fact that the “blitzes” were held at the conveniently located ND HPACT clinic, and HUD-VASH case workers were able to drive those who needed transportation, provided logistical advantages that CA Veterans lacked. For example, CA5, who is visually impaired, relied solely on public transportation and reported other logistical barriers that prevented him from getting vaccinated despite being willing. Similarly, other studies have found that individuals experiencing homelessness cited access and transportation barriers to receiving a COVID-19 vaccine [9, 49]. Mobile clinics conducting outreach in shelters, housing programs, and on the streets where homeless persons are more likely to reside have been shown to reduce these barriers and improve care continuity and adherence [53–55].

Previous studies have found that housing insecurity was associated with a decreased odds of COVID-19 vaccination [5, 7, 56–58] and other care-seeking behaviors [19, 25, 33, 53, 59], as individuals experiencing homelessness often prioritize housing, food, and employment concerns over their health needs. COVID-19 vaccine uptake was lower among HPACT Veterans in this study (50%) than among GPD-enrolled Veterans interviewed in a previously published study (60%) [14]. The unsheltered status of nearly half of the CA HPACT Veterans in this study, as compared to Veterans residing in GPD facilities, likely drives this disparity between the two groups. Being unsheltered, without a constant place to stay, seemed to lead Veterans to lack the interest, resources, and opportunity to vaccinate. GPD program-enrolled Veterans reported experiencing no access barriers to receiving a COVID-19 vaccine, as GPD staff members were facilitating vaccination onsite or were providing transportation to vaccine clinics [13, 14]. In contrast, transportation was cited as a major barrier by CA HPACT Veterans, all of whom relied on their own personal transport. Enrollment in residential programs like GPD, where staff members can engage Veterans in conversations about vaccines, likely offers the types of “high touch” personal approaches most effective in facilitating acceptance [60]. Indeed, housing programs often act as a crucial support system during disasters [61, 62]. Thus, expanding eligibility and increasing enrollment into these existing programs will likely improve health behaviors among Veterans and individuals experiencing homelessness [26, 63–67].

Routine access to care [5, 7, 68] and positive perceptions of care [25, 29, 69] are typically associated with health-seeking behaviors. However, Veterans experiencing homelessness often report negative prior care experiences [15], which can influence receipt of future care and lead to health inequity [25, 29, 70]. While negative experiences were reported by only a minority of Veterans in this study, those in the CA HPACT reported challenges navigating the broader health care system, which may have been due to the clinic’s significantly higher caseload and the size of their VA health care facility, as CA5 and CA13 mentioned. Veterans’ high level of satisfaction with their HPACT experiences at the ND site are likely factors in their high vaccination rates, whereas Veterans who were unvaccinated, at both sites, were more likely to report negative experiences with healthcare and the VA. Additionally, Veterans who received a flu shot in the last two years and Veterans in CA who were medically at-risk were more likely to be vaccinated, suggesting that those with more frequent contact with the medical system may either be more trusting of vaccination or have a greater sense of urgency to vaccinate due to their higher risk.

Nearly all Veterans reported that they trusted the information about COVID-19 vaccines that was given to them by their HPACT clinicians, supporting findings that health care providers are typically one of the most trusted sources of information about COVID-19 [8, 9, 49, 60, 68, 71–73]. However, the Veterans who stated that they trusted their healthcare providers included vaccine refusers, suggesting that trust is necessary but not sufficient to motivate vaccine uptake. CA Veterans were less likely than their ND counterparts to report receiving educational information about the COVID-19 vaccines from their HPACT providers. Notably, CA5 indicated not being notified by the VA that the vaccine was available when he reported being willing to get vaccinated, and lack of coordination between providers left him believing that vaccination would cause medical complications due to his impending surgery. Two of the three vaccinated CA Veterans only accepted the vaccine after months of persuasion, which suggests a higher level of hesitancy overall, underscoring the level of care, effort and attention needed to convince many CA HPACT Veterans to get vaccinated, a significant challenge at a large HPACT clinic. Lastly, distrustful views expressed by CA Veterans who refused the vaccine (CA16, 17, and 18) may have made HPACT providers reluctant to engage further, possibly explaining their reported non-receipt of vaccine information from providers (CA18), or their providers not pursuing the topic, saying, “*it’s up to you*” (CA 16 and 17). HPACT providers at the CA site have reported dropping the vaccination topic upon encountering reluctance among Veteran patients to avoid disengagement or refusal of care [13].

These findings have several implications for health care system planning for individuals experiencing homelessness during health emergencies. First, the care capacity and size of the two HPACT clinics profoundly impacted their vaccine uptake rates. ND HPACT’s smaller, more intimate clinic enabled the primary care provider to establish a rapport of trust and empathy, as reported by several ND Veteran patients and corroborated by another HPACT provider in the same clinic [13]. This ability to empathetically listen, which helps reduce stigma and enables Veterans to feel heard and valued [74], is key to the culturally competent health care [75] that is essential to HPACT’s objective of addressing barriers that often hinder Veterans experiencing homelessness from accessing care [25, 27]. ND5, who was vaccinated early despite believing in conspiracy theories, praised the individualized empathy and care the ND HPACT provider offered. This relationship was more challenging to attain in the significantly larger CA HPACT, where primary care clinicians served 500–700 patients each [13]. While many CA Veterans said they received adequate care from their HPACT, CA5, an unsheltered Veteran with multiple medical conditions, reflected that “*nobody mails you information or calls you from the VA*”, suggesting that such Veterans may not receiving as much individual attention to their needs as would be possible given smaller caseloads. However, CA HPACT providers indicated that they conducted systematic educational outreach to encourage vaccination among Veterans, including both phone calls offering to answer questions, and mailed information [13]. Possibly, CA5’s combined visual impairment and unsheltered status during the initial vaccine rollout made it especially difficult to reach out to this newly enrolled Veteran. Reducing patient caseloads and improving provider patient staffing ratios may help boost vaccine acceptance rates by developing the types of personalized care appeals that succeed in motivating hesitant Veterans.

Second, while the influence of conspiracy theories on vaccine hesitancy is not surprising given that misinformation has been a global phenomenon shown to hinder vaccine acceptance in many national and local contexts [9, 16, 60], these Veterans’ narratives reveal that they are simultaneously highly aware of the stigma attached to “conspiracy theories” and eager to disavow that label for themselves. Several asserted, quite strongly, that they are “*not conspiracy theorists*” while promoting beliefs that would widely be labeled as conspiracy theories, dismissing prominent political commentators on a conversative television media outlet, and claiming to adhere to reputable government sources of data such as the CDC and the VAERS database. Conspiracy theory adherence is often associated with distrust in government authorities, who are generally the public face of COVID-19 vaccine promotion in the U.S. [45–47]. Cynicism and government distrust are pervasive within narratives among Veterans in VA homeless programs [28], creating fertile ground for beliefs in conspiracies. These vaccine-reluctant Veterans may be wedded to an identity of self-reliance given their mistrust of mainstream news information [16], that could stem in part from their military culture [28, 76]. This finding suggests that the most effective messengers to vaccine hesitant Veterans should avoid directly discrediting or criticizing Veterans’ vaccine beliefs, but rather offer factual unbiased information to counter actively circulating misinformation as suggested in the literature [77–79]. Trusted sources of information, particularly health care and housing providers, social workers, and peers will be especially vital [13, 14, 80].

### Limitations

This study is a comparative case study of Veterans in two very different HPACT clinic settings, and its findings may not be generalizable to Veterans’ experiences in other HPACT clinics. The smaller ND clinic had a single primary care provider who was able to offer personalized care to the 110 Veterans in her care; however, such experiences may not be easily replicable nor scalable with larger caseloads. It is also possible that the ND’s clinic’s positive experiences in overcoming community misinformation and vaccine hesitancy were a product of that provider’s uniquely empathetic personality and may not be as easily replicated in other regions with high levels of misinformation or vaccine hesitancy. Further, the CA HPACT clinic also represents an extreme in portraying a region with very high levels of unsheltered homelessness and high primary care caseloads. Even if it were possible to increase the number of providers to offer more personalized patient care, doing so without addressing the epidemic of unsheltered homelessness in some communities in the U.S. West Coast may not markedly increase adherence to vaccination or other desired health behaviors among HPACT Veterans, given other access barriers.

This research, taken in combination with prior studies that found higher vaccine uptake among VA GPD-enrolled Veterans [7, 14] and even among HUD-VASH-enrolled Veterans [7], sheds light on the unique challenges of unhoused Veterans’ who may be unsheltered or whose only “touch” with social services is with a VA health care clinic. These Veterans are likely to be more reflective of all Veterans experiencing homelessness, particularly in urban areas where unsheltered Veterans represent half of the total unhoused Veteran population [44]. More structured research, with a larger number of HPACT sites, would help identify the key factors determining such differences in vaccination rates between HPACT clinics, enabling public health entities, health care providers, and other social services to improve vaccine acceptance among this population.

## Conclusion

The delay or refusal of vaccination among people experiencing homelessness has hindered the ability of members of this vulnerable population to protect themselves from their disproportionate risk of COVID-19 transmission, morbidity, and mortality. Narratives from Veterans experiencing homelessness about what it takes for them to trust a health care provider, or their resistance to having their vaccine concerns be labeled as “conspiracy theories”, often yields insights into what is required to improve care [70], which can expand adherence to recommended protective health behaviors like vaccination. This and other research [14] suggest that improving access to health care and housing for Veterans experiencing homelessness are effective tools for increasing their receptivity and uptake of the COVID-19 vaccine by increasing their contact with trusted messengers. Exploring vaccination attitudes and behaviors, as was done in this study, should enable health systems and homeless service providers to better tailor their care to the needs of people experiencing homelessness during future health emergencies.

### Electronic supplementary material

Below is the link to the electronic supplementary material.

Supplementary Material 1

## Data Availability

The datasets generated and/or analyzed during the current study are not publicly available. They are available from the corresponding author on reasonable request, subject to approval from the ethics committee that approved the study.

## Abbreviations

CA: California
CBOC: Community-Based Outpatient Clinics
CDC: U.S. Centers for Disease Control and Prevention
CRRC: Community Resource and Referral Centers
CTRS: Care, Treatment, and Rehabilitative Service
GPD: Grant and Per Diem
HPACT: Homeless Patient Aligned Care Teams
HUD-VASH: U.S. Department of Housing and Urban Development (HUD) Veterans Affairs Supportive Housing
ND: North Dakota
VA: U.S. Department of Veterans Affairs
VAERS: Vaccine Adverse Event Reporting System
VAMC: VA Medical Center
