# Supplementary material for: COVID-19 vaccination uptake and receptivity among veterans enrolled in homelessness-tailored primary health care clinics: provider trust vs. misinformation
Source: BMC Prim Care. 2024 Jan 12;25:24. doi: 10.1186/s12875-023-02251-x (PMC10785369; doi:10.1186/s12875-023-02251-x)
Supplement: Supplementary file 1 — Supplementary Material 1 [file 12875_2023_2251_MOESM1_ESM.docx]

**COVID Vaccination Uptake among Veterans Enrolled in the**

**VA Homeless Patient Aligned Care Team (H-PACT)**

**Homeless HPACT COVID-19 VACCINATION INTERVIEW GUIDE PHASE 1**

**INTRODUCTION**

*Hi, I’m June Gin, the lead on this study. I’ll be conducting the interview today. First, do you have any questions before we start?*

*I’ll start by giving you some information about our research study. We’re from the Veterans Emergency Management Evaluation Center (VEMEC)* *within the VA. We’re excited to talk with you today because we’re very interested in your opinions on the Covid-19 vaccines. We’re doing these interviews to help the VA deliver better care. All information that we collect will remain anonymous and confidential to the fullest extent allowed by law. We won’t disclose any information about you, and nor the fact that you participated in this study. Participation in this project is entirely voluntary and will not affect your access to VA services. You may decline to answer any question or stop participating at any point. The entire interview should take about 30 minutes. We are interested in your experiences and opinions. Do you have any questions before we begin with our questions?*

***Verbal Consent:*** *“Thank you for agreeing to participate in this interview. Before we begin, it is necessary that I get your verbal consent to audiotape this interview. This recording will only be used for this study. Do I have your consent to audiotape you during this interview?”.*

**BACKGROUND**

1. **You were invited to participate in this study because you receive health care from the VA’s HPACT program. Can you tell us about how long you’ve been in the HPACT program and how your experience with HPACT has been?**

**COVID-19**

*Now I would like to shift gears to speak about the Covid-19.*

1. **How worried are you about Covid-19? (Could include getting infected, getting sick, or dying)**
2. **Can you tell me some of the things that make you (worried/not worried) about Covid-19?**
   1. During the COVID-19 pandemic, how have you felt about the safety of your living situation? Have you been concerned about getting infected?
3. **How likely are you to get the Covid-19 vaccine when it becomes available to y ou?**

**On a scale of 1-5, where 1 is Definitely not; 2 is Probably not; 3 is Don’t know/Neutral/Don’t have enough info; 4 is Probably Yes; 5 is Definitely yes.**

1. **What are your reasons for feeling this way?**
2. IF YES, possible prompts could be: Advice from health care provider; Protection against severe illness; Possible pre-existing conditions among self or others
3. IF NO, possible prompts could be: Worry about the side effects; Too busy; Lack of trust in government; Don’t like shots, discomfort)
4. **[IF WILLING TO GET THE VACCINE] If you were to try to get the Covid-19 vaccine, do you anticipate having any difficulties with the process? For example:**
   1. Prompts: Time, technology access (cell phone or internet for appt), availability, transportation, work schedule, family responsibilities)
   2. Would you be more willing to get the vaccine if you only needed one dose rather than two? [prompt: Johnson and Johnson vaccine only requires one dose, whereas the Pfizer and Moderna vaccines that require two doses, would this influence your willingness?]
   3. Does your willingness to get the vaccine depend on where you would get it? For example, would you be more willing to get it if you could get it at the VA?

**INFORMATION SOURCES AND ATTITUDES**

*Now we would like to ask you some questions about sources of information about the COVID vaccine*

1. **Where have you received most of your information about the Covid-19 vaccine?**
   1. prompt on TV, internet, radio, newspapers, friends/family
2. **Has your HPACT health care provider talked with you about the Covid-19 vaccine?**
   1. Have they recommended that you get the Covid-19 vaccine?
   2. What kind of information did you receive from your health care provider; what did they tell you?
   3. Do you generally trust the information you get from your VA health care provider?
   4. Have you ever felt disrespected or mistreated by a health care provider (e.g. in regard to your appearance, your education, cultural background, etc.), enough so that you considered not returning to that health care facility?
3. **[ASK IF DON’T WANT VACCINE] What else, if anything, might ease your worry and concerns about getting the vaccine?**
   1. If you don’t want the vaccine now or are not sure, what would have to change for you to decide to get the vaccine?
   2. Are there any sources of information that would make you more willing to get the vaccine?

**MEDICAL CARE**

*Next, we would like to ask you a couple of questions about your medical care.*

1. **Did you get a flu shot either during this current flu season or the year before?**
   1. IF YES: Where do you usually receive it?
   2. Did your health care provider recommend that you get the flu shot?
2. **Could you tell us what your reasons for (receiving/not receiving) the flu vaccine in these past years?**
3. **[ASK ONLY IF HESITANT] How do you feel about vaccines in general? (Example: the flu shot)**
   1. Have you ever been reluctant or hesitated to get a vaccination?
   2. Have you ever refused a vaccination? If yes, which vaccine(s) did you refuse?­­­­­­­­­­­­­­­­­­
4. **Do you consider yourself to be in a medically high-risk category, due to your age or any health conditions you might have? Yes or No?**
   1. E.g. obesity, heart disease, diabetes, high blood pressure, asthma, lung disease (COPD), older adults

**SOCIODEMOGRAPHIC INFORMATION**

*If it’s okay with you, we would like to ask a couple of questions about your housing situation and demographics. If you feel uncomfortable about answering any of these questions, please feel free to tell us that you would like to skip any or all of the following questions.*

1. **What is your age? Feel free to give us an age range. Are you in your 30s, 40s, 50s, 60s, etc.?**
2. **Which of the following best describes you?**
   1. Asian or Pacific Islander
   2. Black or African American
   3. Hispanic or Latino
   4. Native American or Alaskan Native
   5. White or Caucasian
   6. Multiracial or Biracial
   7. Race/ethnicity not mentioned
3. **In the last 30 days, where did you stay overnight most frequently?**

1= In my own place (home, rented apartment, or rented room)

2= Transitional housing or residential treatment program (GPD, CERS, Domiciliary, Health Care for Homeless Veterans, etc.): ________________________________________________________

3= Doubled up (staying with friends or family, not on lease) Ask if couch-surfing or have their own room___________________________________________

4= Shelter (please specify): ____________________________

5= Street

6= Other (please specify):______________________________

7= Unknown

**CLOSING**

1. **Is there anything else that we haven’t asked you today, that you’d like to share with us, about your opinions of the Covid-19 vaccines?**

*Thank you for taking the time to speak with us. And thank you so much for your service to our country.*

*Would it be alright for us to follow up with you again in a few months, to check in with you for a 15-minute interview, and learn more about your thoughts about the Covid-19 pandemic and your thoughts about the vaccination process?*
